# Inhibitory control mediates the interaction between serotonin transporter gene (*5-HTTLPR*) and peer victimization on adolescent depressive symptoms

**DOI:** 10.1038/s41598-021-94267-5

**Published:** 2021-07-19

**Authors:** Xiaonan Lin, Yanmiao Cao, Linqin Ji, Wenxin Zhang

**Affiliations:** Department of Psychology, Shandong Normal University, No. 88 East Wenhua Road, Jinan, 250014 Shandong Province China

**Keywords:** Psychology, Human behaviour

## Abstract

Many efforts have been devoted to investigating the effect of the interaction between the serotonin transporter gene (*5-HTTLPR*) and environment (G × E) on depression, but they yield mixed results. The inconsistency has suggested that G × E effects may be more complex than originally conceptualized, and further study is warranted. This study explored the association among *5-HTTLPR*, peer victimization and depressive symptoms and the underlying mediating role of inhibitory control in this association. A total of 871 Chinese Han adolescents (*M*_age_ = 15.32 years, 50.3% girls) participated and provided saliva samples from which the *5-HTTLPR* was genotyped. This study found that *5-HTTLPR* interacted with peer victimization in predicting depressive symptoms. Adolescents carrying L allele reported more depressive symptoms than SS carriers when exposed to higher level of peer victimization. Furthermore, adolescents’ inhibitory control deficits mediated the association between *5-HTTLPR* × peer victimization and depressive symptoms. These findings suggested that one pathway in which G × E may confer vulnerability to depressive symptoms is through disruptions to adolescents’ inhibitory control system.

## Introduction

Depression is a common mental health problem that usually has its initial onset during adolescence. Adolescent depression is related to various mental and physical health problems, such as chronic illness, suicides, and substance abuse^1,2^, hence, it is important to examine both its antecedents and the underlying mechanisms.

Ample evidence has suggested that stressful interpersonal experience is a critical risk factor for depression. Peer victimization is a common stressful experience among adolescents of being a target of physical, verbal, and relational aggression of others^3^, which has been shown to result in negative self-evaluations, leading to depression^4^. However, adolescents exposed to peer victimization vary widely in their vulnerability to depression^5,6^, because genetic factors influence the degree to which an individual is sensitive to environments.

Since the landmark study by Caspi et al.^7^, the functional polymorphism in the promoter region of the serotonin transporter gene (*5-HTTLPR*) which influences the rate of serotonin transcription, has been frequently studied as a moderator in the associations between stressors and depression^5,6,8^. To our knowledge, despite ample research on *5-HTTLPR* × stress interaction, there are only five studies have investigated the role of the *5-HTTLPR* in the relationship between peer victimization and depressive symptoms and yield mixed results. Three studies found that S allele confers greater vulnerability to the deleterious effect of peer victimization^5,6,8^, one study failed to show a significant interaction^9^, and one study found L allele carriers are more at risk as a result of peer victimization^10^. The meta-analyses also yielded varying results, with some providing support for this G × E effect^11,12^, while others casting doubt on the robustness of G × E effect^13,14^. These mixed findings suggested that G × E effects may be more complex than originally conceptualized, and further study is warranted^12^.

Because of the inconsistency and small effect size of G × E, researchers have despaired of seeking a direct association between G × E and behavioral phenotypes. They turned to a process-oriented approach, which is proposed to describe children’s cognitive, social, emotional, and physiological responses to child developmental outcomes^15^. Adopting this approach in research on the association between G × E and behavioral phenotypes, was to explore the underlying processes by which the G × E is expressed^16^.

Emerging neurobiological research has suggested that one endophenotype as potential mediator is inhibitory control which refers to controlling one’s dominant responses to override a strong internal predisposition or external stimulus^17^. For example, the functional magnetic resonance imaging (fMRI) and electroencephalography (EEG) studies have linked the *5-HTTLPR* × stress to the dorsolateral prefrontal cortex (dlPFC) volumes^18^ and activity^19^ which have been consistently reported to be implicated in the function of inhibitory control^20,21^. In addition, research has documented the abnormalities in the dlPFC in depressed individuals, such as lower gray matter^22^ and reduced activity of dlPFC^23^. Besides, according to the cognitive model of depression, inhibitory control deficits contribute to impaired disengagement from negative thoughts or biased attention, which in turn triggers depression^24,25^. Thus, taken together with the preceding neuroimaging evidence, inhibitory control may bridge the gaps from *5-HTTLPR* × stress interactions to depression. Furthermore, the limited empirical research supported the notion that genes and environments may confer vulnerability to depression through inhibitory control deficits^26,27^. Knyazev et al.^26^ found that expressive suppression—a related trait of executive functioning—mediated the effect of *5-HTTLPR* by stress interaction on depression, with S allele conferring more vulnerable to stress.

In summary, the current study was to investigate the moderating role of *5-HTTLPR* in the relation between peer victimization and adolescent depressive symptoms in Chinese Han sample. More importantly, adopting the process-oriented approach the current study aimed to explore whether inhibitory control functioning would mediate the influence of *5-HTTLPR* and peer victimization on adolescent depressive symptoms.

## Results

### Preliminary analyses

The genotype distribution for *5-HTTLPR* (*n* = 10, LL; *n* = 237, SL; *n* = 624, SS) was consistent with Hardy–Weinberg equilibrium (*p* = 0.06). Owing to the frequency of the LL genotype is very low in our sample (*n* = 10), and such a small sample was insufficient for analysis, in line with previous studies with Asian population^28,29^, the LL and SL genotypes were collapsed into an L allele group and coded as 1, SS genotype was coded as 0, to maximize the power of the analyses. There was no gender difference in genotype frequencies (χ^2^ = 4.67, *d* = 2, *p* = 0.10). Gender was dummy coded into 0 (girls) and 1 (boys).

Means, standard deviations and correlations for all main variables were presented in Table 1. Both peer victimization and inhibitory control deficits showed significant positive correlations with depressive symptoms. The *5-HTTLPR*, however, was not associated with depressive symptoms and inhibitory control deficits. Peer victimization was positively related to inhibitory control deficits. No significant gene-environment correlation (*r*GE) between *5-HTTLPR* and peer victimization was observed (*t* = − 0.23*, p* = 0.82). In addition, there was no significant gender difference in depressive symptoms (*t* = − 0.10, *p* = 0.92), but boys reported higher level of peer victimization (*t* = − 6.32, *p* < 10^–6^) and inhibitory control deficits (*t* = − 3.86, *p* = 1.2 × 10^–4^) than girls.

**Table 1:** Means, standard deviations, and correlations among the primary variables.

	*M*	*SD*	Gender^a^	*5-HTTLPR*^a^	Peer victimization^b^	Inhibitory control	Depressive symptoms
1. Gender^a^	–	–	1	− 0.05	0.21***	0.14***	0.00
2. *5-HTTLPR*^a^	–	–		1	0.01	0.00	0.02
3. Peer victimization^b^	0.33	0.40			1	0.12***	0.41***
4. Inhibitory control	0.24	0.28				1	0.14***
5. Depressive symptoms	0.27	0.25					1

*p < 0.05; ** *p* < 0.01; ***p < 0.001.

^a^*5-HTTLPR* genotype and Gender were dummy coded: SS = 0, L = 1; Girls = 0 and Boys = 1.

^b^Peer victimization was standardized.

### Primary analysis I: *5-HTTLPR* × peer victimization

**Figure 1:**
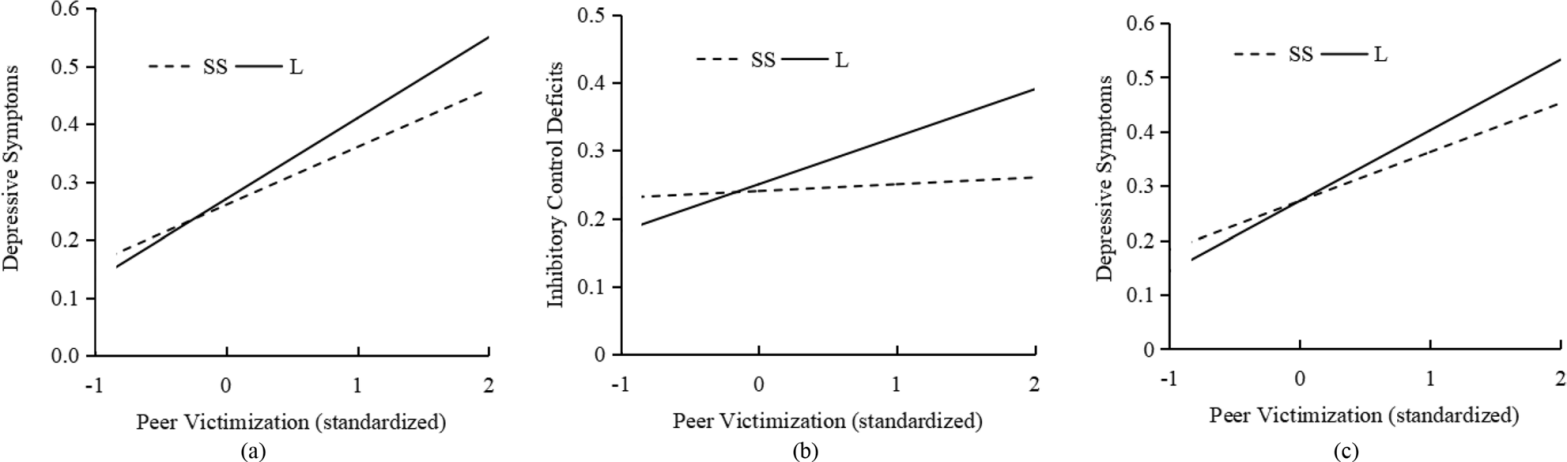
Graphical plots of interaction between peer victimization and *5-HTTLPR* in the prediction of depressive symptoms (**a**), inhibitory control deficits (**b**), and depressive symptoms (when added inhibitory control deficits to model as a mediated variable) (**c**). *Solid line*, L allele carriers; *Dash line,* SS homozygotes carriers.

The results of Model 1 were shown in Table 2. The main effect of peer victimization was significant in predicting adolescent depressive symptoms (*b* = 0.11, *p* < 10^–6^), while no significant effect of *5-HTTLPR* was found (*b* = 0.00, *p* = 0.81). The *5-HTTLPR* × peer victimization interaction was significant (*b* = 0.05, B-H corrected *p* = 0.012). Simple slope analyses indicated that, compared to SS carriers (*b* = 0.11, *p* < 10^–6^), peer victimization was more likely to predict depressive symptoms for L allele carriers (*b* = 0.14, *p* < 10^–6^; see Fig. 1a).

**Table 2:** Test of mediated moderation.

	Model 1 (Depressive symptoms)						Model 2a (Inhibitory control)						Model 2b (Depressive symptoms)					
	*ΔR*^*2*^	*b*	β	*t*	*p*	95% CI^a^	*ΔR*^2^	*b*	β	*t*	*p*	95% CI^*d*^	*ΔR*^2^	*b*	β	*t*	*p*	95% CI^a^
**Step 1**																		
Gender	0.00	0.00	0.00	00.10	0.92	[− 0.03, 0.04]	0.02***	**0.08**	**0.14**	**3.89**	**1.1** × **10**^**–4**^	**[0.04, 0.11]**	0.00	0.01	0.02	0.44	0.66	[− 0.03, 0.04]
**Step 2**																		
PV	0.18***	**0.11**	**0.43**	**13.73**	** < 10**^**–6**^	**[0.09, 0.13]**	0.01*	**0.03**	**0.10**	**2.78**	**0.01**	**[0.00, 0.05]**	0.17***	**0.10**	**0.40**	**12.24**	** < 10**^**–6**^	**[0.08, 0.12]**
*5-HTTLPR*		0.00	0.01	0.24	0.81	[− 0.03, 0.04]		0.01	0.01	0.29	0.78	[− 0.03, 0.05]		− 0.00	− 0.00	− 0.06	0.95	[− 0.03, 0.03]
IC														**0.09**	**0.10**	**3.17**	**0.002**	**[0.03, 0.16]**
**Step 3**																		
G × E	0.01**	**0.05**	**0.10**	**2.68**	**0.008**	**[0.00, 0.09]**	0.01**	**0.07**	**0.13**	**3.19**	**0.001**	**[0.02, 0.13]**	0.00	0.04	0.07	1.92	0.055	[− 0.01, 0.08]
*R*^*2*^		0.19***						0.04***						0.18***				
*F*		49.31***						8.38***						34.70***				

Significant results are highlighted by bold face.

*PV* peer victimization (standardized); *IC* inhibitory control; *G* × *E 5-HTTLPR* × peer victimization.

^a^Bootstrapping based confidence intervals.

*p < 0.05; **p < 0.01; ***p < 0.001.

### Primary analysis II: the mediation role of inhibitory control deficits

As shown in Table 2, in Model 2a, no significant effect of *5-HTTLPR* was found in predicting adolescent inhibitory control deficits (*b* = 0.01, *p* = 0.78), while the main effect of peer victimization (*b* = 0.03, *p* = 0.01) and the *5-HTTLPR* × peer victimization interaction (*b* = 0.07, B-H corrected *p* = 0.003) were significant. Simple slope analyses showed that peer victimization predicted inhibitory control deficits among individuals carrying L allele (*b* = 0.05, *p* = 0.03) but not among SS homozygotes (*b* = − 0.01, *p* = 0.62; See Fig. 1b).

In Model 2b, inhibition deficits was positively related to depressive symptoms (*b* = 0.09, *p* = 0.002). However, it should be noted that, when inhibition was included in the model, the effect of G × E on depressive symptoms was not significant (*b* = 0.04, B-H corrected *p* = 0.055), but the interactive patterns exhibited very similar trends with those observed in Model 1. Compared to adolescents carrying SS genotype (*b* = 0.11, *p* < 10^–6^), adolescents carrying L allele reported more depressive symptoms when experience higher level of peer victimization (*b* = 0.13, *p* < 10^–6^; see also Fig. 1c). Further bootstrapping tests indicated that inhibitory control deficits mediated the association between *5-HTTLPR* × peer victimization and depressive symptoms (95% CI [0.00, 0.02], *R*^2^ = 0.18, *p* < 10^–6^).

### Supplemental analyses: internal replication

To test the robustness of these findings, an internal replication analysis was conducted by randomly splitting the total sample into two subsamples. None of the primary variables (i.e., gender, χ^2^ = 0.03, *p* = 0.87; *5-HTTLPR*, χ^2^ = 0.65, *p* = 0.42; peer victimization, *t* = − 0.47, *p* = 0.64; inhibitory control deficits, *t* = − 0.48, *p* = 0.82; depressive symptoms, *t* = − 0.08, *p* = 0.94) were significantly different between two subsamples.

The results showed that the G × E (Table 2, Model 1 and Model 2a) was replicated in subsample 1 (*b* = 0.07, B-H corrected *p* = 0.04; *b* = 0.10, B-H corrected *p* = 0.006; see Table 3) but not in subsample 2 (*b* = 0.03, B-H corrected *p* = 0.28; *b* = 0.06, B-H corrected *p* = 0.069; see Table 4). Model 2b was not replicated in both subsamples (*b* = 0.06, B-H corrected *p* = 0.06; *b* = 0.01, B-H corrected *p* = 0.67; see Tables 3, 4). However, Bootstrapping tests indicated that the indirect effects from *5-HTTLPR* × peer victimization to depressive symptoms via inhibitory control deficits were significant in both subsamples, with the indirect paths between *5-HTTLPR* × peer victimization and depressive symptoms significantly different from zero in both subsamples (subsample1: 95% CI [0.00, 0.03], *R*^2^ = 0.15, *p* < 10^–6^; subsample 2: 95% CI [0.00, 0.02], *R*^2^ = 0.21, *p* < 10^–6^).

**Table 3:** Test of mediated moderation in subsample 1.

	Model 1 (depressive symptoms)						Model 2a (inhibitory control)						Model 2b (depressive symptoms)					
	*ΔR*^*2*^	*b*	β	*t*	*p*	95% CI^a^	*ΔR*^*2*^	*b*	β	*t*	*p*	95% CI^a^	*ΔR*^*2*^	*b*	β	*t*	*p*	95% CI^a^
**Step 1**																		
Gender	0.00	0.01	0.02	0.41	0.68	[− 0.04, 0.05]	0.02*	**0.07**	**0.12**	**2.49**	**0.01**	**[0.02, 0.12]**	0.00	0.02	0.03	0.64	0.52	[− 0.03, 0.06]
**Step 2**																		
PV	0.15***	**0.10**	**0.40**	**8.76**	** < 10**^**–6**^	**[0.07, 0.13]**	0.03**	**0.05**	**0.16**	**3.17**	**0.002**	**[0.01, 0.09]**	0.14***	**0.09**	**0.35**	**7.29**	** < 10**^**–6**^	**[0.06, 0.12]**
*5-HTTLPR*		0.00	0.01	0.13	0.89	[− 0.04, 0.05]		0.02	0.04	0.72	0.47	[− 0.04, 0.08]		0.00	0.00	0.00	0.99	[− 0.05, 0.05]
IC														**0.10**	**0.12**	**2.48**	**0.01**	**[0.01, 0.19]**
**Step 3**																		
G × E	0.01*	**0.07**	**0.12**	**2.45**	**0.02**	**[0.00, 0.13]**	0.02**	**0.10**	**0.16**	**2.96**	**0.003**	**[0.01, 0.19]**	0.01*	0.06	0.11	2.13	0.03	[− 0.01, 0.13]
*R*^*2*^		0.16***						0.06***						0.15***				
*F*		20.99***						6.45***						14.43***				

Significant results are highlighted by bold face.

*PV* peer victimization (standardized); *IC* inhibitory control; *G* × *E 5-HTTLPR* × peer victimization.

*p < 0.05; ** *p* < 0.01; *** *p* < 0.001.

^a^Bootstrapping based confidence intervals.

**Table 4:** Test of mediated moderation in subsample 2.

	Model 1 (depressive symptoms)						Model 2a (inhibitory control)						Model 2b (depressive symptoms)					
	*ΔR*^*2*^	*b*	β	*t*	*p*	95% CI^a^	*ΔR*^*2*^	*b*	β	*t*	*p*	95% CI^a^	*ΔR*^*2*^	*b*	β	*t*	*p*	95% CI^a^
**Step 1**																		
Gender	0.00	− 0.01	− 0.01	− 0.27	0.79	[− 0.05, 0.04]	0.02**	**0.08**	**0.15**	**3.03**	**0.003**	**[0.03, 0.13]**	0.00	0.00	− 0.00	− 0.01	0.99	[− 0.05, 0.05]
**Step 2**																		
PV	0.21***	**0.12**	**0.47**	**10.61**	** < 10**^**–6**^	**[0.09, 0.14]**	0.00	0.01	0.04	0.75	0.46	[− 0.02, 0.04]	**0**.21***	**0.12**	**0.45**	**9.95**	** < 10**^**–6**^	**[0.09, 0.14]**
*5-HTTLPR*		0.00	0.01	0.11	0.92	[− 0.05, 0.05]		− 0.01	− 0.01	− 0.21	0.83	[− 0.06, 0.05]		− 0.00	− 0.01	− 0.17	0.87	[− 0.05, 0.05]
IC														**0.09**	**0.10**	**2.13**	**0.03**	**[0.01, 0.18]**
**Step 3**																		
G × E	0.00	0.03	0.06	1.22	0.23	[− 0.03, 0.08]	0.01*	0.06	0.12	2.01	0.046	[0.00, 0.12]	0.00	0.01	0.02	0.43	0.67	[− 0.05, 0.08]
*R*^*2*^		0.21***						0.03**						0.21***				
*F*		28.74***						3.46**						21.06***				

Significant results are highlighted by bold face.

*PV* peer victimization (standardized); *IC* inhibitory control; *G* × *E 5-HTTLPR* × peer victimization.

*p < 0.05; **p < 0.01; ***p < 0.001.

^a^Bootstrapping based confidence intervals.

## Discussion

Although there has been significant interest in exploring the *5-HTTLPR* by environment interactions on depression, the extant studies to identify the G × E have been hampered by inconsistency^13,14^, requiring the question to be explored in greater depth. Recently, researchers have sought to make more progress in understanding the inconsistent G × E by exploring the underlying mechanisms of how genes and environments operate to influence depression. Therefore, following the process-oriented perspective, we explored whether *5-HTTLPR* interacts with peer victimization in predicting adolescent depressive symptoms and further investigated the underlying mechanism.

There was a significant *5-HTTLPR* × peer victimization in predicting adolescent depressive symptoms. Specifically, adolescents carrying L allele were at increased risk for developing depressive symptoms when exposed to higher level of peer victimization, compared to SS carriers, indicating that the L allele confers vulnerability to depressive symptoms in the face of adversity. However, in previous studies the S allele has been identified as a vulnerable genotype^5,8^. One possibility behind the inconsistencies in *5-HTTLPR* related findings may be racial differences across studies. Such racial differences have also been observed in fMRI studies on the associations between *5-HTTLPR* and the response of the amygdala to fearful stimuli^30,31^. Based primarily on Caucasian samples, Hariri et al.^30^ reported greater amygdala hyperreactivity in response to fearful stimuli among S allele carriers compared to LL individuals in two independent samples. However, in a sample of 38 Chinese Han participants, Li et al.^31^ found that L allele was associated with amygdala hyperactivation. It has been established that amygdala hyperactivation is a risk factor for depression^32^. Therefore, the L allele for Chinese adolescents may be associated with higher risk for depressive symptoms by virtue of its association with increased amygdala activity. However, it is unclear that why the same functional alleles act in different ways in the associations with brain activity or depression in Caucasian and Chinese populations. To our knowledge, the underlying mechanisms explaining the differential association between *5-HTTLPR* and depression in different races remain unclear at present. Williams et al.^33^ pointed that the racial differences in linkage disequilibrium (LD) between the *5-HTTLPR* and other unknown genes may result in a reversal of the effect of vulnerable genotype on the sensitivity to environment. However, because the present study does not assess other genes in LD with *5-HTTLPR,* we cannot directly test this possibility. The findings in current study need to be interpreted with caution until replicated, and further investigations are needed.

We found that adolescent inhibitory control deficits mediated the interaction between *5-HTTLPR* and peer victimization in predicting depressive symptoms. Peer victimization was a significant predictor of worse inhibitory control deficits only for L allele carriers. Inhibitory control deficits, in turn, predicted adolescent depressive symptoms. Although the indirect effect was only modest, these findings are consistent with prior research documenting the *5-HTTLPR* × stress interaction on abnormalities in the dlPFC^18,19^ and the relation between dlPFC and depression^34^. These results can also be interpreted from the late-maturing PFC theory of adolescent depression^35^, that is, the weaker top-down control of PFC known to support inhibitory control, failed to inhibit activation in emotional brain regions, which in turn results in depression. Evidence suggested that *5-HTTLPR* L allele is associated with decreased functional connectivity between amygdala and PFC among Chinese Han sample^29^. Thus, gene related abnormalities in PFC-amygdala connectivity may render adolescents particularly vulnerable to negative environments by reducing the capacity of PFC to inhibit hyperactivation in amygdala aroused by peer victimization.

Besides, supplemental analysis revealed that the direct association between G × E and depression was not fully replicated, but the mediating mechanisms were relatively robust. These results implied that the G × E were more detectable and robust at the level of relatively more biologically based phenotypes than behavioral phenotypes. Exploring the underlying mechanisms of G × E is therefore one promising attempt to understand the nature of G × E.

The present study has several strengths. First, this study adopted the process-oriented approach in understanding the inconsistency of G × E, which provided preliminary evidence for the hypothesis of gene-endophenotype-behavior. Second, a large sample size provided greater statistical power in exploring the G × E. Despite several strengths, it is important to consider the limitations of this study. First, the cross-sectional design does not allow making causal inferences. Although the theoretical considerations and prospective studies provided substantial support for this association, future research with longitudinal design will be needed to fully explore the mediated moderation model. Second, this study did not assess the minor allele rs25531, which comprises a single-nucleotide variant (A → G) within the L allele that renders an L_G_ allele functionally similar to the S allele^36^. Besides, existing literature is mixed in regards to heterozygote (SL) grouping because the function of the SL genotype is less understood. Due to the low frequency of LL genotype, it is insufficient for considering all three genotypes separately in this study. To better understood the nature of G × E interactions, various genotype grouping approaches (S-dominant, L-dominant, additive, and two dummy variables) should be considered in future studies. Third, this study did not conduct an external replication because of the lack of samples with the same measures. Fourth, inhibitory control was assessed by the mother-report questionnaire, which is prone to informant biases and inaccuracies in retrospective recall. Duku and Vaillancourt^37^ reported low cross-informant agreement of BRIEF in younger children. Thus, these results may lie in a possible observational bias, and should be interpret with caution.

## Conclusion

This study provided evidence for *5-HTTLPR* × peer victimization interaction. Results suggested that L allele carriers may be more affected by negative experience of peer victimization than SS carriers, and consequently had more depressive symptoms. Moreover, the present study showed the value in taking a gene-endophenotype-behavior perspective when understanding the mechanism by which the G × E operate to influence psychopathology outcomes such as depression. Specifically, in line with the cognitive model of depression, the risk alleles and adversities may confer vulnerability to depression by undermining the function of inhibitory control. These results may also be helpful in developing targets of treatment and therapeutic tools for these adolescents, especially inhibitory control regulation techniques.

## Methods

### Participants

One thousand and ninety 9^th^ grade adolescents were originally recruited from 12 middle schools in the urban areas of Jinan, capital city of Shandong province in eastern China. Among the 1090 participants, 156 adolescents did not attend school due to illness, death, changing schools etc. during the period of data collecting. As a result, the final sample were 934 adolescent-mother dyads (response rate was above 85%; *M*_*age*_ = 15.32 years, *SD* = 0.49 years, 50.3% girls).

To minimize potential population stratification bias, the analyses were conducted on the sample of Chinese Han ethnicity (*n* = 904). No significant differences were found between adolescents of Chinese Han and Chinese minorities in terms of gender (χ^2^ = 0.001, *df* = 1, *p* = 0.97), age (*t* = − 0.21, *p* = 0.84), genotype distribution (χ^2^ = 0.39, *df* = 2, *p* = 0.82), peer victimization (*t* = − 0.96, *p* = 0.34), inhibitory control deficits (*t* = − 0.94, *p* = 0.35), and depressive symptoms (*t* = 0.65, *p* = 0.55). Additional, thirty-three participants were excluded for missing more than 50% of primary variables including *5-HTTLPR*, peer victimization, inhibitory control deficits, and depressive symptoms. Excluded participants (*n* = 33) did not differ from remaining participants (*n* = 871, *M*_*age*_ = 15.31 years, *SD* = 0.49 years, 51.2% girls) in terms of age (*t* = 1.11, *p* = 0.28), but with more boys (*t* = 2.97, *p* = 0.01).

### Procedures

This study was approved by the ethics committee of Shandong Normal University. All procedures of the study were in accordance with the ethical standards of the ethics committee of Shandong Normal University on human experimentation and with the 1964 Helsinki declaration and its later amendments or comparable ethical standards. Informed assent from adolescents themselves and consent from their parents and school principals were obtained in advance of data collection.

Adolescents were asked to provide their saliva samples for DNA extraction and complete a series of questionnaires. After data collection, each participates involved acquired a gift (about $1). When adolescents finished the self-report questionnaires, they were given an envelope to take home. The adolescents’ parents (72.3% cases were mothers, *n* = 630) completed the questionnaires in the envelope regarding adolescents’ inhibitory control deficits, and the students took the envelope back to their head teachers the following day.

### Measures

#### Depressive symptoms

The Children’s Depression Inventory (CDI)^38^ was used to assess adolescent depressive symptoms. The Chinese version of CDI was modified and proved to be reliable and valid by Chen and Li^39^. The CDI consists of 27 items (e.g.,“I am sad all the time”). For each item, the participants identified one of the three statements that best described themselves during the past two weeks. Higher mean scores indicate more symptoms. The Cronbach’s α was 0.88 in this study.

#### Peer victimization

Peer victimization was assessed via a self-report questionnaire, which adapted from the Multidimensional Peer Victimization Scale (MPVS)^40^. This questionnaire is a useful tool with satisfactory reliability and validity for assessing peer victimization in Chinese adolescents^41^. The altered version of the scale includes 14 items and was used to assess adolescents’ experience of physical (e.g., “Other kids hurt me physically in some way”), relational (e.g., “Other kids made other people not talk to me”), and verbal (e.g., “Made fun of me because of my appearance”) forms of peer victimization. Using a 4-point scale from 0 (never) to 3 (often), adolescents reported the frequency with which each experience occurred during the current semester. The responses were averaged to form a continuous total score of victimization, with higher score indicating higher level of victimization. The Cronbach’s α for this study was 0.92.

#### Inhibitory control deficits

The subscale of 10 items from the Behavior Rating Inventory of Executive Function (BRIEF)^42^ was used to assess inhibitory control in everyday situations via mother-report (e.g., “gets out of control more than friends.”). The questionnaire has been proved to have good ecological validity and reliability under the background of Chinese culture^43^. The participants answered on a 3-point scale from 1 (never) to 3 (often) and higher averaged score indicated higher level of inhibitory control deficits. The Cronbach’s α was 0.81 in this study.

#### Genotyping

Genomic DNA was extracted from participants’ saliva samples. DNA extraction and genotyping were performed using the MALDI-TOF in the MassARRAY system (Sequenom Inc., San Diego, California, USA) according to the manufacturer’s instructions. The *5-HTTLPR* polymorphism was amplified using the following primer sequences: forward 5’- GGCGTTGCCGCTCTGAATTGC-3’ and reverse 5’-GAGGGACTGAGCTGGACAACCCAC-3’. Genotype calling was performed with MassARRAY RT software 3.0.0.4 and analyzed using the MassARRAY Typer software 3.4.

#### Statistical analyses

According to the step-by-step instructions for testing the mediated moderation^44^, three regression models were conducted. First, we used a moderated model (i.e., Model 1) to test the association between depressive symptoms and *5-HTTLPR* genotype, peer victimization, and their interaction. Second, we examined whether the *5-HTTLPR* genotype moderated the relationship between peer victimization and inhibitory deficits (i.e., Model 2a). Third, the *5-HTTLPR*, peer victimization, inhibitory control deficits, and the G × E interaction were included as predictors in the final regression model (Model 2b). To control Type I error, the corresponding *p* level was corrected using the Benjamini and Hochberg (B-H) procedure^45^. In each model, significant interactions were further investigated using simple slope analyses. The bootstrapping tests using the SPSS macro PROCESS (http://www.afhayes.com) suggested by Hayes^46^ were also conducted to test the mediated moderation effect. Adolescents’ gender was included as a covariate in each model. All continuous predictors were standardized before analyses to avoid multicollinearity. To test the robustness of results, the internal replication analyses were conducted by randomly split the total sample into two subsamples. All above analyses were performed in SPSS 20.0.

## Data Availability

Due to privacy or ethical restrictions, the data that support the findings of this study are not publicly available, but can be obtained on reasonable request from the corresponding author.
